# Dynamic changes in perivascular space morphology predict signs of spaceflight-associated neuro-ocular syndrome in bed rest

**DOI:** 10.1038/s41526-024-00368-6

**Published:** 2024-03-01

**Authors:** Sutton B. Richmond, Rachael D. Seidler, Jeffrey J. Iliff, Daniel L. Schwartz, Madison Luther, Lisa C. Silbert, Scott J. Wood, Jacob J. Bloomberg, Edwin Mulder, Jessica K. Lee, Alberto De Luca, Juan Piantino

**Affiliations:** 1 Department of Applied Physiology and Kinesiology, University of Florida, 1864 Stadium Rd., Gainesville, FL USA; 2 Norman Fixel Institute for Neurological Diseases, University of Florida, Gainesville, FL USA; 3 Department of Psychiatry and Behavioral Sciences, University of Washington School of Medicine, Seattle, WA USA; 4 Department of Neurology, University of Washington School of Medicine, Seattle, WA USA; 5 VISN 20 Mental Illness Research, Education and Clinical Center (MIRECC), VA Puget Sound Health Care System, Seattle, WA USA; 6 Layton-NIA Oregon Aging and Alzheimer’s Disease Research Center, Department of Neurology, Oregon Health & Science University, Portland, OR USA; 7 Advanced Imaging Research Center, Oregon Health & Science University, Portland, OR USA; 8 Department of Pediatrics, Division of Child Neurology, Doernbecher Children’s Hospital, Oregon Health and Science University, Portland, OR USA; 9 Veteran’s Affairs Portland Health Care System, Neurology, Portland, OR USA; 10 NASA Johnson Space Center, Houston, TX USA; 11 German Aerospace Center (DLR), Cologne, Germany; 12 Department of Radiology, University Medical Center Utrecht, Utrecht, Netherlands

**Keywords:** Health care, Pathogenesis, Signs and symptoms

## Abstract

During long-duration spaceflight, astronauts experience headward fluid shifts and expansion of the cerebral perivascular spaces (PVS). A major limitation to our understanding of the changes in brain structure and physiology induced by spaceflight stems from the logistical difficulties of studying astronauts. The current study aimed to determine whether PVS changes also occur on Earth with the spaceflight analog head-down tilt bed rest (HDBR). We examined how the number and morphology of magnetic resonance imaging-visible PVS (MV-PVS) are affected by HDBR with and without elevated carbon dioxide (CO_2_). These environments mimic the headward fluid shifts, body unloading, and elevated CO_2_ observed aboard the International Space Station. Additionally, we sought to understand how changes in MV-PVS are associated with signs of Spaceflight Associated Neuro-ocular Syndrome (SANS), ocular structural alterations that can occur with spaceflight. Participants were separated into two bed rest campaigns: HDBR (60 days) and HDBR + CO_2_ (30 days with elevated ambient CO_2_). Both groups completed multiple magnetic resonance image acquisitions before, during, and post-bed rest. We found that at the group level, neither spaceflight analog affected MV-PVS quantity or morphology. However, when taking into account SANS status, persons exhibiting signs of SANS showed little or no MV-PVS changes, whereas their No-SANS counterparts showed MV-PVS morphological changes during the HDBR + CO_2_ campaign. These findings highlight spaceflight analogs as models for inducing changes in MV-PVS and implicate MV-PVS dynamic compliance as a mechanism underlying SANS. These findings may lead to countermeasures to mitigate health risks associated with human spaceflight.

## Introduction

The perivascular transit passageway comprises a brain-wide network of perivascular spaces (PVS) that maintains brain homeostasis by removing or redistributing metabolic products, inflammatory and immune-mediated molecules, and additional solutes^1^, providing an exit pathway along the draining veins^2–5^. PVS enlargement visible on magnetic resonance imaging (MRI) has been proposed as an indirect marker of impaired solute clearance and glymphatic dysfunction^6–11^. We recently reported that novice astronauts (first-time flyers) displayed a significant pre- to post-flight increase in MRI-visible PVS (MV-PVS) volume compared to more experienced (i.e., one or more prior spaceflights) crewmembers. This finding suggests a potential holdover effect or loss of MV-PVS compliance from previous spaceflight (s)^12^. Another recent study reported MV-PVS enlargement and an association between MV-PVS volume and ventricular dilatation in astronauts undergoing spaceflight^13^. It remains unclear whether MV-PVS enlargement results in any adverse outcome or represents a compensatory response to microgravity. Studies on the effects of spaceflight on cerebral fluid dynamics and their potential clinical implications are limited by the small sample of astronauts and the inability to scan participants during a mission. Thus, determining whether such changes are replicated in spaceflight simulations would allow for a better understanding of these phenomena.

Head-down tilt bed rest (HDBR) is commonly used in spaceflight simulation experiments. HDBR results in body changes similar to those provoked by microgravity during spaceflight, including a fluid shift towards the head and compression of the top of the brain against the skull^14–18^. Participants undergoing HDBR may also be exposed to increased carbon dioxide (CO_2_) levels (i.e., hypercapnia) to simulate the enclosed environment of the International Space Station (ISS)^19^. Hypercapnia increases cerebral blood flow, arterial blood pressure^20^, and MV-PVS volume^21^. In addition to fluid shifts, nearly half (~45%) of participants undergoing hypercapnic HDBR in the Visual Impairment Intracranial Pressure and Psychological :envihab Research (“VaPER”) campaign experienced signs of spaceflight-associated neuro-ocular syndrome (SANS)^22^. SANS consists of optic disc edema, choroidal folding, globe flattening, and hyperopic refractive error shifts and has been reported in as high as 75% of people returning from long-duration spaceflights^23^. The etiological mechanisms behind SANS remain insufficiently understood. Headward fluid shifts, increased intracranial pressure, folate status, altered fluid drainage, hypercapnia-related volume, and pressure disturbances have been proposed as etiologies of SANS^24–31^. Of note, there are greater pre- to post-flight changes in white matter-MV-PVS volumes in astronauts who developed signs of SANS^13^, supporting altered fluid drainage via PVS enlargement as a potential underlying mechanism of SANS. These potential mechanisms underlying the enlargement of PVS in space crews have been reviewed by Wostyn et al. (2022), where the authors speculate that the dilation of PVS observed in long-duration space travelers leads to a dysregulation of the brain and ocular glymphatic systems^28^.

Based on these observations, this is an exploratory study aimed at assessing the effects of 30 days of HDBR and hypercapnia on MV-PVS morphology. We performed a secondary analysis of MRI data collected from the NASA partnership with the German Space Agency VaPER campaign, 2017-18. We predicted increased white matter MV-PVS number, volume, length, and/or width with long-duration HDBR. Based on prior observations^13^, we also predicted greater increases in white matter MV-PVS number and size for the bed rest participants that exhibited signs of SANS versus those that did not. VaPER participants were compared to a cohort of participants who also underwent HDBR but no hypercapnia, the Artificial Gravity Bed Rest Study with European Space Agency (“AGBRESA”) campaign, 2019, a joint study between NASA, the European Space Agency, and the German Aerospace Center^32–34^. We predicted that individuals exposed to HDBR and elevated CO_2_ would exhibit greater MV-PVS morphological changes than those undergoing HDBR with ambient air conditions^13^. As a last aim of this assessment, we sought to identify how the combination of HDBR and elevated CO_2_ affects the diffusion tensor imaging along the perivascular space (DTI-ALPS) index^35^. The DTI-ALPS index has been proposed as a surrogate marker of glymphatic fluid flow along periventricular perivascular spaces^35–37^; however, whether it is a valid marker of cerebral solute clearance remains unclear. If the DTI-ALPS index and MV-PVSs both reflect changes in perivascular fluid dynamics, we predict that the DTI-ALPS index will a) be directly correlated to MV-PVS volume and number and b) decrease with HDBR + CO_2_.

## Results

Serial T1 scans were collected from eleven healthy, young, male and female participants who underwent 30 days of strict 6° HDBR with elevated CO_2_ (group: HDBR + CO_2_) and eight control (no hypercapnia) participants who underwent 60 days of strict 6° HDBR breathing ambient air (group: HDBR; *see* Table 1*for demographics*). Each cohort was imaged multiple times prior to, during, and after bed rest (HDBR + CO_2_: day −13, −7, 7, 29, +7, + 13 and HDBR: day −13, −7, 29, 58, +10). Of the nineteen participants examined, five individuals, all within the HDBR + CO_2_ cohort, developed signs of SANS.

**Table 1:** Demographic and Anthropometric Summary Means ± Standard Deviations

	HDBR (*n* = *8*)	HDBR + CO_2_ (*n* = *11*)	*p*-value
Age (years)	34 ± 8	34 ± 8	0.85
Gender *Female: Male* (n)	2:6	5:6	-
Height (m)	1.8 ± 0.1	1.7 ± 0.1	0.45
Weight (kg)	79.4 ± 12.7	70.8 ± 8.6	0.09
Body Mass Index (kg^.^m^−2^)	25.2 ± 2.6	23.4 ± 2.2	0.13
SANS Present *Female: Male* (n)	0:0	3:2	-
HDBR Duration (days)	60	30	-
Elevated CO_2_ (Yes / No)	No	Yes	-

Demographic and anthropometric differences between groups were compared using Student independent t-tests. No significant demographic and anthropometric differences between groups were identified. *SANS* Spaceflight-Associated Neuro-Ocular Syndrome, *HDBR* Head-Down Tilt Bed Rest, *CO*_*2*_ Carbon dioxide.

### No changes in MV-PVS characteristics pre- to post-HDBR

Changes in MV-PVS characteristics and DTI-ALPS index with HDBR and HDBR + CO_2_ are summarized in Supplementary Table 1. There were no statistically significant (*p* > 0.05; Supplementary Table 2) group-level changes in any of the MV-PVS characteristics from pre- to post-HDBR, nor were there group-by-time interactions when comparing ambient air to elevated CO_2_ participants (Supplementary Table 2).

### Group differences in MV-PVS morphology by SANS in HDBR + CO_2_

**Fig. 1:**
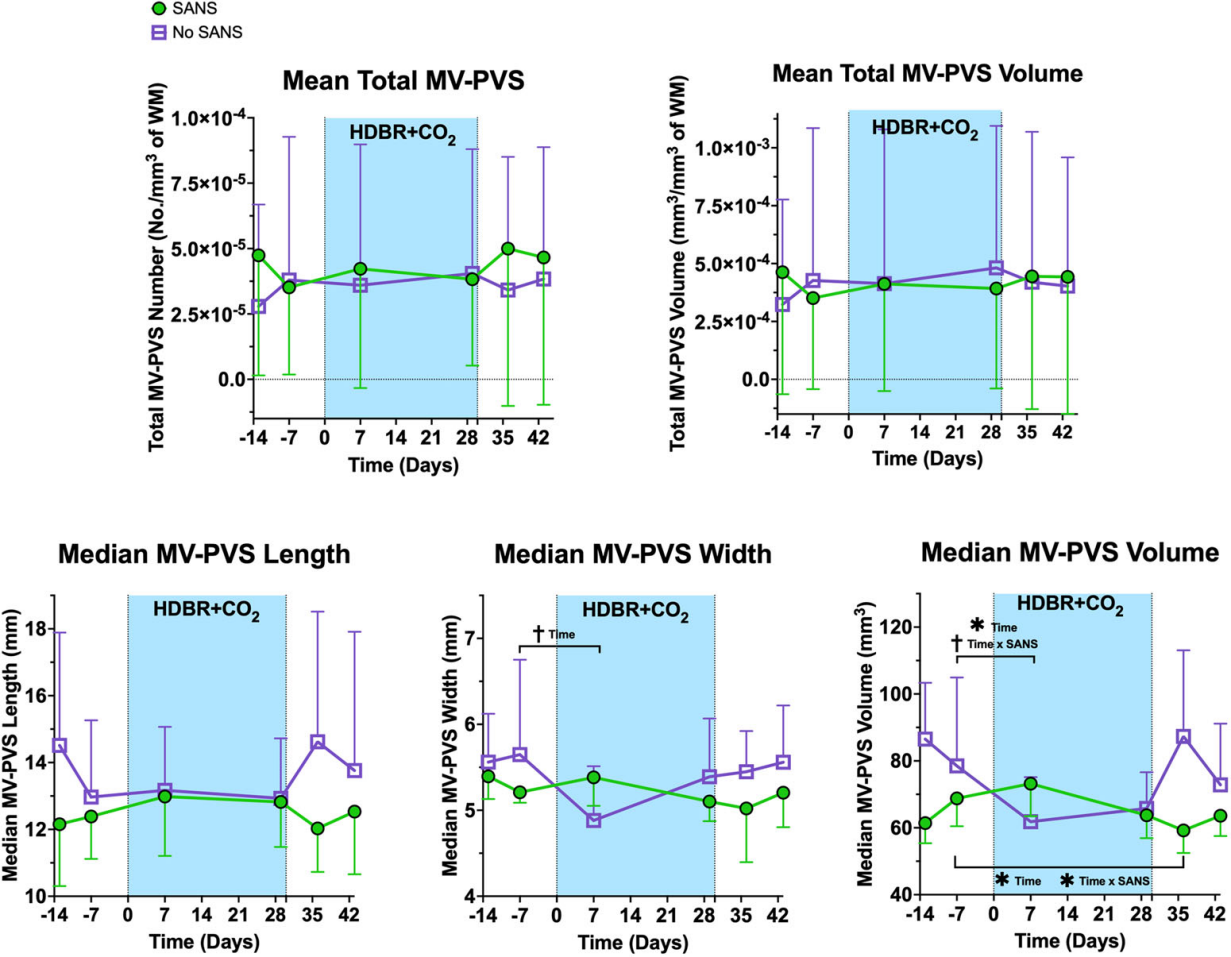
Changes in MV-PVS metrics from pre- to post-bed rest for persons with signs of SANS compared to those without. The HDBR + CO_2_ data are split into SANS (green) and No-SANS (purple) subgroups. Bars represent standard deviation. The width of the blue box indicates the duration of HDBR + CO_2_. *Indicates a statistically significant (*p* < 0.05) group difference between the SANS and No-SANS participants for changes in median MV-PVS volume and diffusivity along the PVS with bed rest. ^**†**^Indicates a statistical trending (*p* < 0.10) change in median MV-PVS width and median MV-PVS volume from pre- to post-bed rest. Significant changes in recovery were examined in instances where significant pre- to post-bed rest changes occurred (i.e., median MV-PVS volume). *SANS* spaceflight-associated neuro-ocular syndrome, *MV-PVS* magnetic resonance imaging-visible perivascular space, *WM* white matter.

**Fig. 2:**
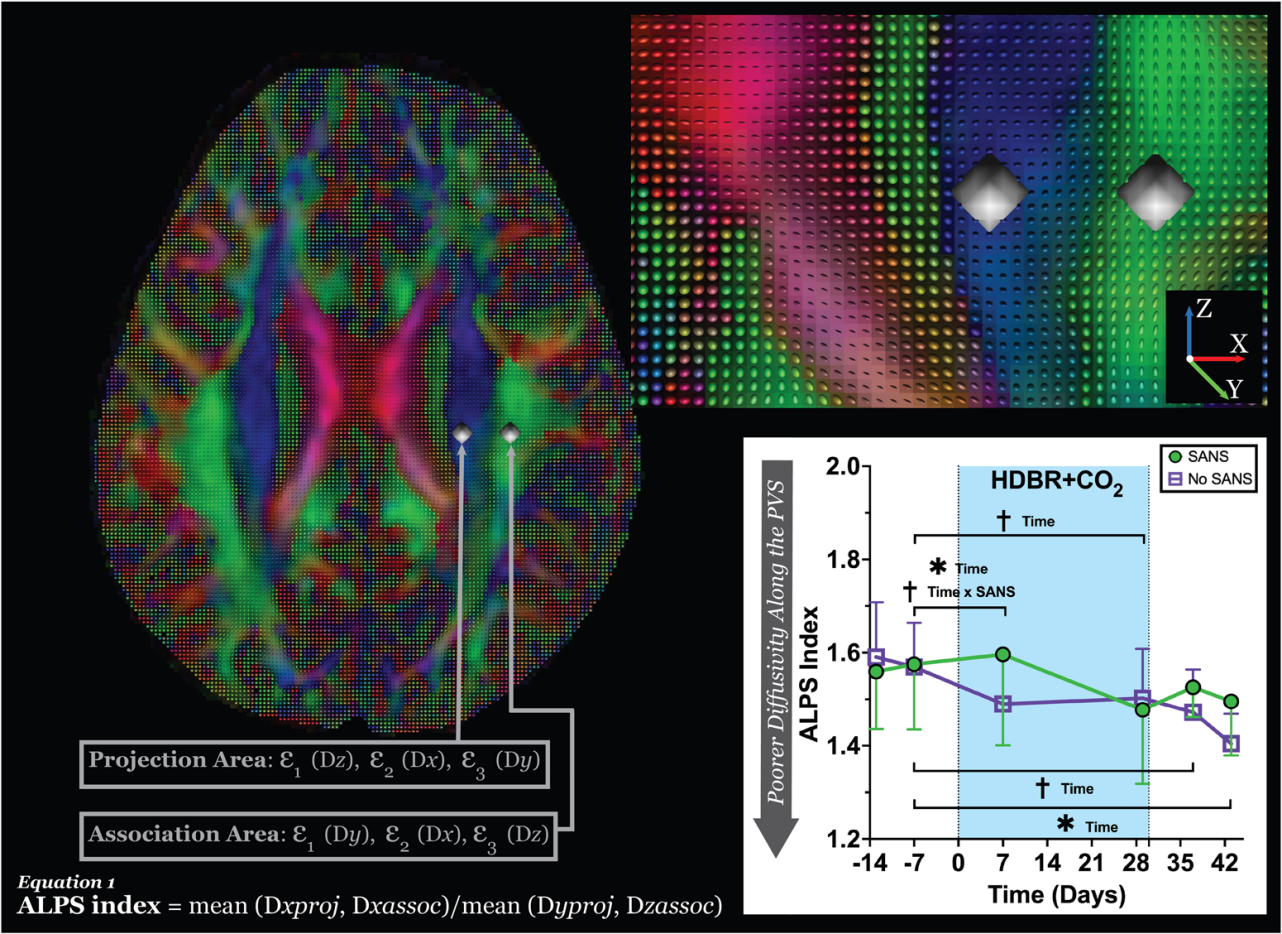
Diffusion tensor image analysis along the perivascular space (DTI-ALPS) methodology. The DTI color map with accompanied glyphs shows the primary (*ε*_1_) eigenvector (scaled by FA) overlaid in the direction of the projection (blue; z-axis) and association (green; y-axis) fibers. The spherical ROIs are placed in the location of the projection and association fibers to measure the diffusivities in these locations and the level of lateral ventricles. The secondary (*ε*_2_) and tertiary (*ε*_3_) eigenvectors for each location compose the DTI-ALPS index, describing the diffusivity occurring along the perivascular space. In the plot, * indicates a statistically significant (*p* < 0.05) group difference between the SANS and No-SANS participants for changes in median MV-PVS volume and diffusivity along the PVS with bed rest. ^**†**^Indicates a statistical trending (*p* < 0.10) change in DTI-ALPS from pre- to post-bed rest. Significant changes in recovery were examined in instances where significant pre- to post-bed rest changes occurred for DTI-ALPS. *SANS* spaceflight-associated neuro-ocular syndrome, *MV-PVS* magnetic resonance imaging-visible perivascular space, *DTI-ALPS* along the perivascular space, *(ε)* eigenvalue.

MV-PVS were identified and characterized in each participant with a multi-step algorithm^38^, determining the structural features of the MV-PVS, including median length, width, and volume, in addition to quantity and volume per mm^3^ of white matter. There were no statistically significant changes in any MV-PVS characteristics from pre- to post-HDBR + CO_2_ (*p* > 0.05; Supplementary Table 3). However, persons undergoing HDBR + CO_2_ who showed signs of SANS exhibited changes in MV-PVS characteristics and DTI-ALPS index compared to those who did not, as summarized in Supplementary Table 3, Figs. 1, and 2. Among those who showed signs of SANS, the median MV-PVS volume decreased significantly compared to 7 days pre-HDBR + CO_2_ (*p* = 0.046). That is, participants that developed signs of SANS exhibited an inverted-U profile, contrasting the trend of their No-SANS counterparts. A time (i.e., 7 days pre- vs. 7 days post-HDBR + CO_2_) by group (SANS) interaction, however, did not reach our pre-determined statistical significance level (*p* = 0.084); Supplementary Table 3. Finally, median MV-PVS volume was significantly lower in the SANS cohort compared to their No-SANS counterparts at seven (*p* = 0.007) and thirteen (*p* = 0.005) days following HDBR + CO_2_, compared to pre-bed rest measures; additionally, there was a significant group (SANS) by time (7 days post-HDBR + CO_2_) interaction (*p* = 0.023) in recovery relative to pre-HDBR + CO_2_ levels; Supplementary Table 4. SANS participants exhibited a concave outline from pre- to post-HDBR; although the groups converged at the culmination of the bed rest protocol, they narrowly returned to their respective pre-bed rest median MV-PVS volume levels by day seven of recovery (Fig. 1).

### Group differences in DTI-ALPS index by SANS during HDBR + CO_2_

There were no statistically significant changes in DTI-ALPS index from pre- to post- HDBR + CO_2_ (*p* > 0.05; Supplementary Table 1). However, the DTI-ALPS index was significantly (*p* = 0.026) different from pre-bed rest to day seven HDBR + CO_2_, narrowly displaying a statistically significant difference by the end of bed rest (29 days post-HDBR + CO_2_ (*p* = 0.057, Fig. 2)). Additionally, an interaction approaching significance (*p* = 0.054) was present seven days into bed rest, where participants who did not show signs of SANS exhibited reduced DTI-ALPS index from pre-bed rest, while SANS participants displayed an elevation in DTI-ALPS index (Fig. 2 and Supplementary Table 1). Seven days into recovery, those who developed signs of SANS demonstrated a higher diffusivity DTI-ALPS than their pre- HDBR + CO_2_ measures (*p* = 0.073) and a significant difference thirteen days post (*p* = 0.005); Fig. 2 and Supplementary Table 2, exhibiting a concave outline, the inverse of their counterparts.

### Group differences in MV-PVS by SANS status combining cohorts

**Fig. 3:**
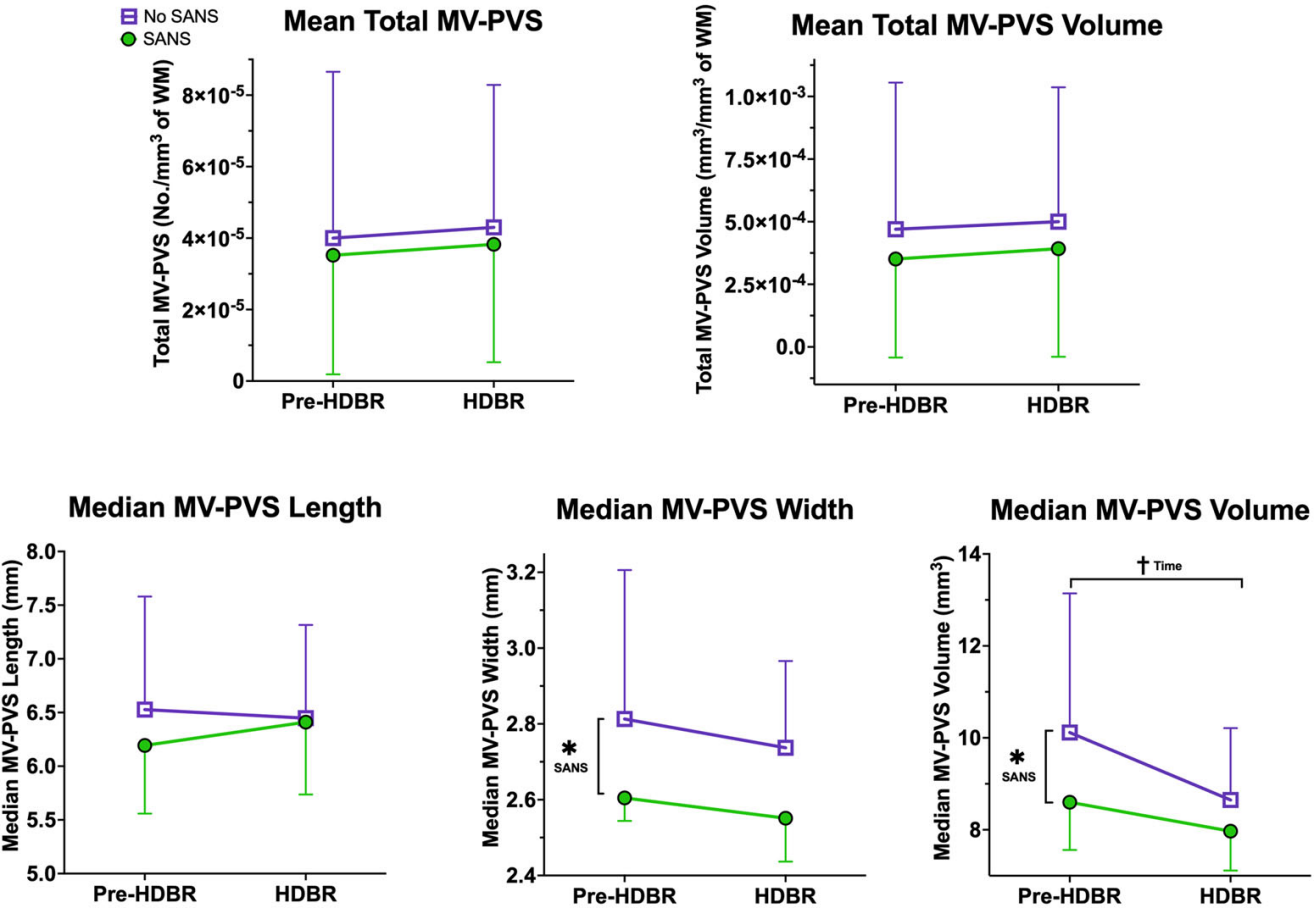
Changes in MV-PVS metrics with bed rest in SANS versus No-SANS. Here in each plot, we depict the group average MV-PVS characteristics for the total sample (HDBR (controls) and HDBR + CO_2_), with error bars representing the standard deviation. The data are split into SANS (green) and No-SANS (purple) subgroups. *Indicates a statistically significant (*p* < 0.05) group difference between the SANS and No-SANS participants for changes in median MV-PVS width and volume with bed rest. ^**†**^Indicates a statistical trending (*p* < 0.10) time effect for median MV-PVS volume from pre- to post-bed rest. *SANS* spaceflight-associated neuro-ocular syndrome, *MV-PVS* magnetic resonance imaging-visible perivascular space, *WM* white matter.

Participants from the two campaigns (VaPER: HDBR + CO_2_, and AGBRESA: HDBR, no CO_2_) were combined (total *n* = 19) and stratified by SANS status. MRI scans obtained 7 days pre-HDBR and 29 days during HDBR overlapped between experimental setups. Therefore, only those two timepoints were used for analysis. In the combined cohort, 5 participants exhibited signs of SANS and 14 did not. Participants who showed signs of SANS had significantly smaller median MV-PVS width (*p* = 0.007) and volume (*p* = 0.034) than their No-SANS counterparts at day 29 compared to 7 days pre-HDBR (Fig. 3). Additionally, across all participants, there was an effect of time in decreasing median MV-PVS volume that approached significance (*p* = 0.078) with bed rest.

## Discussion

In this exploratory, small sample investigation, we examined whether there are morphological variations in MV-PVS with HDBR and whether changes differ in participants who do versus do not show signs of SANS. Overall, we identified that exposure to long-duration HDBR does not alter MV-PVS significantly. Further, we determined that the combined effects of HDBR + CO_2_ did not result in significant changes in MV-PVS characteristics compared to HDBR alone, contradicting our initial hypotheses. However, in the HDBR + CO_2_ cohort, the participants that did not display signs of SANS had a significant reduction in median MV-PVS volume with bed rest compared to their SANS counterparts, opposing our initial prediction. Additionally, the No-SANS subgroup also presented a reduced DTI-ALPS index with HDBR + CO_2_.

We previously reported that astronauts did not show a significant pre- to post-flight change in MV-PVS, unless we compared novice (first time) versus experienced flyers^12^. Another study found group level changes in MV-PVS with spaceflight^13^. One of the observable effects of spaceflight is the appearance of SANS in certain individuals. To date, there are mixed findings as to whether brain changes are linked to SANS and, further, if these changes could extend to space flight analogs (i.e., HDBR). Lee et al.^38^ reported no significant alterations in white matter, gray matter, or free water dispersion in HDBR participants affected by SANS^38^. On the other hand, our previous work identified differential changes in visual cortical connectivity and behavioral effects^39^ in those exhibiting signs of SANS. In line with a study that reported differential changes in MV-PVS with spaceflight in crewmembers that did versus did not show signs of SANS^13^, we found a different MV-PVS response to HDBR in those showing signs of SANS compared to those who did not. HDBR + CO_2_ participants who developed signs of SANS displayed a significantly higher median MV-PVS volume, width, and DTI-ALPS index after entering bed rest than their No-SANS counterparts. Further, SANS participants exhibited marginal fluctuation compared to the participants who did not display signs of SANS across all three MV-PVS measures (Fig. 1, bottom row) a week after entering bed rest. These observations contradict the increase in white matter PVS volumes from before to after long-duration spaceflights in NASA astronauts who developed signs of SANS in comparison to those that did not, reported by Barisano and colleagues^13^. The absence of significant PVS fluctuations post-HDBR in our cohort suggests that additional extreme environment-induced stressors or return travel could be inducing PVS alterations outside the scope of the spaceflight analog design. The underlying mechanism for this difference remains speculative but could be related to sleep quality experienced by astronauts and analog participants. Given that PVS function is associated with sleep quality^40^ and repeated accounts of sleep deprivation have been reported by astronauts^41,42^, it is ostensible that sleep deprivation could be an influential factor in comparing non-terrestrial and terrestrial-based observations. There are also differences in the duration of environmental exposures; current spaceflights are quite a bit longer than typical HDBR studies. We may be capturing faster dynamic changes with HDBR than are currently possible with spaceflight. Future endeavors should assess whether space-induced sleep deprivation and PVS alterations are associated.

In our cohort, participants who developed SANS had smaller MV-PVS at the start of the study. Also, their MV-PVS did not dilate during bed rest compared to participants with no SANS. These observations in the SANS group agree with the predicted effect of tissue unweighting around the vessels^43,44^. The reduction or elimination of compressive forces surrounding the vessels (via microgravity or simulated microgravity) is postulated by Wostyn and colleagues to alter venous hemodynamics and reduce CSF outflow^28^. Impaired CSF outflow is predicted to increase orbital CSF pressure, leading to flattening of the posterior ocular chamber and the hyperopic refractive errors seen in SANS. Under this model, the results from our data set suggest that MV-PVS dilatation may represent a compensatory mechanism, allowing CSF outflow. The role of hypercapnia in SANS is unclear. Some participants in the VaPER campaign developed signs of SANS, but some participants in the AGBRESA campaign—which did not have elevated CO_2_ levels—also did. Laurie et al.^45^ collected data in the VaPER campaign as well and found that there were no changes in arterialized PCO_2_ (partial pressure of CO_2_) at their measurement timepoints. Thus, the role of elevated CO_2_ in the effects observed here is ambiguous. Furthermore, we speculate that the MV-PVS’s ability to respond dynamically to changes in CSF and blood circulation represents a clinically relevant characteristic of these structures. Thus, our data calls into question whether the traditional representation of enlarged MV-PVS as strictly a sign of dysfunction^11,46–52^ remains accurate.

Traditionally, elevated MV-PVS burden or enlargement (e.g., increased volume) has been regarded as indicative of injury^11,46^, health conditions^47^, disease^48–52^, and disruptions to brain homeostasis^53^. However, across the morphological and diffusivity measures in the current study, we observed that those who developed signs of SANS had smaller MV-PVS before going into HDBR and also exhibited no changes, in contrast to their No-SANS counterparts. This observation indicates that the brain’s response to the headward fluid shifts and upward shift of the brain may be predicated on the state prior to the intervention. It may be that those with less compliance in the PVS are those that experience system stress, resulting in signs of SANS. Lower body negative pressure has been evaluated as a potential countermeasure for SANS and headward fluid shifts that occur in microgravity. It reduces inflight intraocular pressure to levels seen on Earth, but has been shown to not be effective at mitigating choroidal thickness^54^. It has not been directly investigated in terms of mitigating perivascular space changes in spaceflight, but has been suggested to underlie differences seen between cosmonauts (who use lower body negative pressure) and astronauts^13^.

Our initial investigation of MV-PVS alterations with spaceflight provided key contributions to understanding how these structures are affected by microgravity; however, spaceflight assessments are limited to pre- and post-mission image acquisitions. This current investigation allowed for more acute observations to be made regarding the effects of spaceflight stressors on brain waste and solute transport. This investigation delivers an evaluation of acute MV-PVS alterations via short time scale interventions, providing depictions of acute reversibility in the MV-PVS morphology of participants without injury or disease. Moreover, based on our findings, we postulate that MV-PVS morphology is an early marker of SANS. Future investigations need to further delineate if reserve capacities can be identified for various stress-induced challenges to the structure of the MV-PVS, and whether these changes are clinically significant. Furthermore, extending these evaluations beyond thirty or sixty days may provide additional insight concerning spaceflight-induced associations between changes in MV-PVS and signs of ocular alterations. Currently, the bed rest spaceflight analog offers the only feasible way to neuroimage pseudo-in-flight changes and utilize larger sample sizes than what is provided with flight-based investigations.

In addition to assessing the MV-PVS morphology, we conducted an analysis of the diffusion-weighted MRI scans, utilizing an emerging index of “diffusivity along the perivascular space (DTI-ALPS)”^55^. First described by Taoka and collaborators^35^, the DTI-ALPS index has been proposed as a “measure of glymphatic function”. Assessing the DTI-ALPS index across the six-time points in the sample (HDBR + CO_2_), we observed DTI-ALPS index values within the range of previously reported values for neurotypical adults^37^, regardless of SANS status. Unexpectedly, persons who developed signs of SANS in the HDBR + CO_2_ campaign exhibited higher DTI-ALPS index entering bed rest. Similar to MV-PVS morphological alterations, DTI-ALPS index in SANS participants fell below participants exhibiting no signs of SANS by the conclusion of bed rest, suggesting decreased diffusivity changes in response to headward fluid shifts and hypercapnia. In this context, the DTI-ALPS index could provide a more effective identifier for those at increased risk of developing SANS. However, the changes observed in the DTI-ALPS index, in conjunction with changes in MV-PVS burden, in particiapnts with SANS, are thought-provoking. The divergence in the recovery periods and the absence of any significant correlation between changes in these two measures suggests that MV-PVS enlargement and decreased DTI-ALPS index represent distinct biological processes. Moving forward, caution should be exhibited when comparing, contrasting, or making inferences about these measures as surrogate markers of waste clearance.

This exploration has several limitations that should be considered when interpreting the findings. First, the sample size is quite small, which may have led to the lack of statistical differences over time and between groups; however, HDBR studies are difficult and expensive to conduct^56–59^, typically resulting in small sample sizes. PVS alterations between groups were not significant in this exploratory investigation; future studies should employ larger sample sizes to more definitively address this. Second, HDBR and elevated CO_2_ only mimic some aspects of spaceflight (i.e., headward fluid shifts, body unloading, hypercapnia)^60^. Other aspects, such as disrupted sleep and circadian cycles, radiation, and prolonged isolation are not well captured by this analog. However, both elevated CO_2_ and headward fluid shifts have been implicated as mechanisms of SANS, making this analog appropriate for the questions investigated here. Another limitation is that we performed post hoc analyses, meaning that we did not plan ahead to acquire T2 MRI scans. Although T1-weighted images provide accurate auto-identification of perivascular spaces in white matter, the algorithm is optimized to T2-weighted images^61^, and we acknowledge that including T2-weighted images may have enhanced MV-PVS segmentation and characterization in this analysis. Furthermore, the MV-PVS algorithm we used is tailored to white matter MV-PVS segmentation; the algorithm has not been optimized for identifying MV-PVS in the brain’s gray matter. Therefore, to better evaluate the relationship between SANS and changes in PVS, additional MRI sequences (e.g., T2-weighted, heavily weighted T2 imaging, inversion recovery, fast functional MRI, etc.) should be evaluated in future investigations. These potential sequences would aid in the assessment of CSF dynamics and slow vasomotor oscillations to provide a better dynamic range for detecting subtle variations in PVS. And finally, the assessment of the DTI-ALPS index was limited to diffusion-weighted images collected in a single cohort (HDBR + CO_2_), limiting DTI-ALPS to within-group comparisons. Given the within-group results highlighted with this evaluation by SANS status, we believe study outcomes warrant further validation between group investigations pertaining to spaceflight and spaceflight analogs. Lastly, because of the neuroanatomical locations of the small ROIs at the level of the lateral ventricle, it is plausible that ventricular enlargement occurring with headward fluid shifts in this sample^38^ could be influencing this measure locally but not globally. This may also explain the lack of correlation between the DTI-ALPS index and the MV-PVS metrics. However, substantiating this theory is beyond the scope of the current evaluation and should be assessed in future studies.

## Conclusions

Our results indicate that at the group level, acute exposure to HDBR and elevated CO_2_ do not affect MV-PVS morphology. However, when evaluated separately for those who do and do not show signs of SANS, we discovered that persons who develop signs of SANS exhibited decreased MV-PVS plasticity/compliance compared to their No-SANS counterparts. Our exploratory findings suggest that an absence of significant MV-PVS changes accompanying HBDR + CO_2_ reflects reduced compliance or flexibility in the system in those developing SANS. Although MV-PVS morphological alterations may provide an etiological mechanism to SANS, it remains to be determined if this decrease in “MV-PVS compliance” is a deficient compensatory mechanism or some other process. Future evaluations of spaceflight analogs should further address the relationship between SANS and PVS changes. Additionally, as recently observed in ventricular compliance with shorter inter-mission spaceflight intervals^62^, PVS should also be assessed in future investigations.

## Methods

### Participants and facilities

Eleven healthy adult participants (group: HDBR + CO_2_) were recruited to participate in the VaPER study (*see Results section*, Table 1*for demographics*), which comprised 30 days of strict 6° HDBR with elevated CO_2_ (0.5% atmospheric CO_2_ (3.8 mmHg partial pressure of CO_2_), 20.9% oxygen, and 78.6% nitrogen), similar to ambient conditions reported aboard the ISS^19^. An additional eight control participants (group: HDBR; see *Results section*, Table 1) underwent 60 days of strict 6° HDBR breathing ambient air (0.04% atmospheric CO_2_, 20.95% oxygen, and 78.08% nitrogen)^63^ for the AGBRESA study^32^. Past HDBR studies allowed participants to use a small pillow if desired. This meant that the “head down” component was not strictly maintained. In both the VAPER and AGBRESA campaigns, a pillow was not allowed. Thus, head-down tilt was more strictly maintained than in past studies. Additionally, both participant groups received a controlled diet and were restricted to eight hours maximum of daily sleep; all experimental procedures were conducted at the German Aerospace Center’s (Cologne, Germany) :envihab facility. There were no group differences in age, height, weight, or body mass index between the HBDR + CO_2_ and the HDBR participants (*Results section*, Table 1). All participants were considered healthy volunteers, absent of any history of physiological (e.g., smoking, injuries) and psychological risk factors, strict dietary requirements (e.g., vegetarian/vegan), or medications (e.g., seasonal allergies, heartburn, acid reflux or indigestion) that would compromise the integrity of the investigation^32,45^. Specific exclusion risk factors included a history of physiological (e.g., musculoskeletal, ophthalmological, neurological) or psychiatric conditions; cardiovascular dysfunction; metabolic or endocrine disturbances (e.g., diabetes mellitus); blood clotting; pulmonary, sleep, or pain disorders; gastroesophageal reflux; renal stones; or the presence of infectious or inflammatory diseases^32^. The institutional review boards of the University of Florida (Gainesville, Florida, USA), NASA, and the commission of the regional medical association (Ärztekammer Nordrhein) approved the methods used in this study, and all participants provided informed consent before participation. All participants received monetary compensation.

### Experimental procedure and imaging protocol

This investigation was a longitudinal 6° HDBR protocol, conducted in two separate groups (HDBR + CO_2_ and HDBR: see *Results section*, Table 1). We have reported on other brain and behavioral changes occurring throughout these studies^38,39,64–66^. Both groups underwent MRI scanning seven days prior to and on day 29 within HDBR, allowing us to compare data between the two campaigns.

**Fig. 4:**
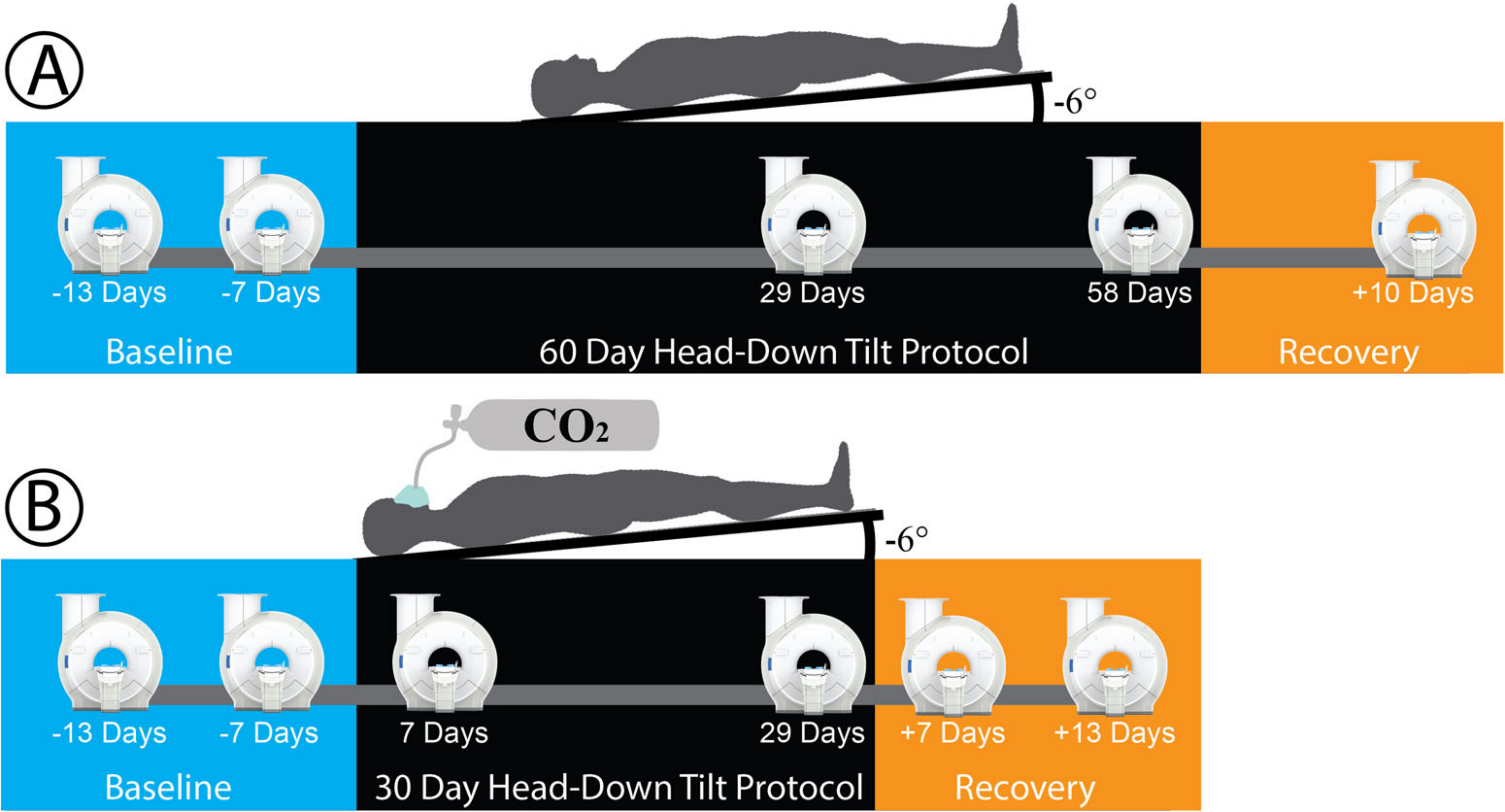
Experimental protocol and MRI acquisition timeline. **A** Testing timeline for the head-down tilt bed rest (HDBR) group, who underwent 60-days of 6° HDBR, breathing ambient air (0.04% atmospheric CO_2_, 20.95% oxygen, and 78.08% nitrogen) concentration. Participants in this group were imaged five times. **B** The HDBR + CO_2_ group was placed in a 6° HDBR position for 30-days, breathing elevated (0.5%) atmospheric CO_2,_ and imaged at six time points.

All participants underwent MRI acquisitions with a 3.0 T Siemens (Siemens Medical Solutions USA, Inc., Malvern, PA) Magnetom Skyra MR scanner before, during, and after the HDBR protocol (*imaging schedule*, Fig. 4). We acquired a T1-weighted gradient echo pulse MPRAGE sequence and a diffusion-weighted sequence (HDBR + CO_2_ only) (*acquisition parameters*, Table 2) while participants were in the supine 6° HDBR position.

**Table 2:** MRI Specifications and Acquisition Parameters

	T1-Weighted Images		Diffusion Weighted Images
	HDBR	HDBR + CO_2_	HDBR + CO_2_^a^
Head/Neck Coil Channels (*n*)	32	16	16
TR (ms)	1900	1900	9800
TE (ms)	2.49	2.44	91.0
Section thickness | Gap (mm)	0.9	1.0	1.0
Flip Angle (°)	9	9	90
Number of Slices (No.)	192	192	49
Voxel Size (mm^3^)	0.94 × 0.94 × 0.94	0.5 × 0.5 × 1.0	1.8 × 1.8 × 2.7
FOV (mm)	270 × 270	250 × 250	235 × 235
Matrix Size (mm)	288 × 288	256 × 256	128 × 128
Diffusion-Unweighted Vol. (*n*)	NA	NA	2
Diffusion-Weighting (s/mm^2^)	NA	NA	1000
Diffusion Directions (*n*)	NA	NA	60
Phase Encode Direction	Ant. ≫ Post.	Ant. ≫ Post.	Ant. ≫ Post.

*HDBR* Head-Down Tilt Bed Rest, *CO*_*2*_ Carbon dioxide, *TR* Repetition Time, *TE* Echo Time.

^a^Diffusion-weighted images were only examined in the HDBR + CO_2_ group. Volumes with no diffusion weighting (*b* = 0 s/mm^2^) were acquired at the beginning of each sampling stream. *Not Applicable* (NA).

However, the head remained flat (0° tilt) during scanning due to MRI head coil constraints.

HDBR participants were imaged five times total, twice prior to the HDBR protocol (13 and 7 days), twice during bed rest (days 29 and 58), and ten days post-bed rest (R + 10) (Fig. 4).

The HDBR + CO_2_ group was imaged at six different time points: twice before bedrest (days -13 and -7), twice during bed rest (days 7 and 29), and twice post-bed rest (recovery (R) days +7 and +13 days) (Fig. 4).

### MV-PVS characterization

Identification and characterization of the MV-PVSs were carried out using techniques described by our group in previous studies^12,61,67^. Briefly, T1-weighted images were skull-stripped (Brain Extraction Tool (BET)^68^ via FMRIB Software Library (FSL; Version 5.0)) and segmented into different tissue types (FreeSurfer, Version 5.1) before undergoing a two-part heterogeneity assessment (*3dLocalstat* and *3dcalc*, Analysis of Functional Neuro Images (AFNI)). Resultant voxels were considered for further assessment if: (1) the voxel resided within the eroded white matter mask, (2) the difference between the voxel’s intensity and the average intensity of the surrounding voxels was > 15%, and (3) the voxel’s intensity fell in the bottom fifth percentile of its neighbors. Subsequent voxels premeditated from voxel clusters > 1 mm^3^ (3D corner-to-corner connectivity, 3dclust type 3, AFNI) were labeled MV-PVSs and quantified with five dependent variables representative of MV-PVS load and characteristics via a custom Matlab (R2016a, MathWorks Inc., Natick, MA) code. For quality assurance, trained raters visually inspected resultant masks for motion degradation, noise degradation, or other artifacts (ML and JP). In total, we excluded two and ten T1-weighted images from further MV-PVS analyses for the VAPeR and AGBRESA participants, respectively. Total MV-PVS volume and count were normalized to individual white matter volumes by dividing the total MV-PVS number and volume by the entire segmented white matter volume (in mm^3^). Thus, white-matter MV-PVS burden was characterized by the (1) MV-PVS volume (mm^3^) per mm^3^ of white matter and (2) MV-PVS number per mm^3^ of white matter. Additionally, for each participant at each time point, the median: (3) volume (mm^3^), (4) length (mm), and (5) width (mm) were used to characterize MV-PVS structures. The use of only T1-weighted images may underestimate MV-PVS burden. However, our method has shown good correlation with visual counts and high inter-class correlation on repeated scans previously^12^, thus providing a reliable estimate of MV-PVS burden.

### Diffusion along the perivascular space

Diffusion-weighted image preprocessing and analysis of diffusion along the perivascular space (DTI-ALPS) index was completed using the ExploreDTI (University Medical Center Utrecht, Netherlands, Version 4.8.6; www.exploredti.com) graphical toolbox^69^ and the FMRIB Software Library’s (FSL) 6.0 toolbox (Analysis Group, FMRIB)^70,71^, and Advanced Normalization Tools (ANTs)^72,73^. Diffusion weighted scans were only assessed in the HDBR + CO_2_ cohort. First, the participant T1-weighted images at all time intervals were realigned to the within-participant first-time point image (i.e., pre-HDBR + CO_2_ (-13 days)), using the FSL MCFLIRT toolbox^74^ image co-registration. After realignment, the baseline image was warped to the Montreal Neurological Institute (MNI) standardized space using ANTs^72,73^; the resultant warp field was applied to the remaining time points. We used a standardized diffusion tensor preprocessing pipeline^75^, where data were inspected for the presence of motion artifact before being corrected for signal drift^76^, Gibbs ringing^77^, subject motion, and eddy currents^78^, as well as distortions^79^ using ExploreDTI^69^. During eddy current/subject motion correction, the resulting images were registered with their T1-weighted images in standard (i.e., Montreal Neurological Institute) space via the ExploreDTI^69^ suite for the region of interest (ROI) placement. Spherical ROIs measuring 4 mm in diameter, originated in FSL, were placed in the regions of the projection and association fibers at the level of the lateral ventricle in the right hemisphere (MNI coordinates: Projection (29, −4, 10) and Association (41, −4, 10); *see* Fig. 2). Tensor-derived eigenvalues from each ROI were plugged into the DTI-ALPS index equation (Fig. 2), described by ref. 35 (see equation 1)^35^.

### Spaceflight-Associated Neuro-ocular Syndrome (SANS)

In addition to recording demographic and anthropometric data, each participant was evaluated for signs of SANS throughout the bed rest protocol with an ophthalmic exam for the presence of: (1) optic disc edema (variable Frisén grades), (2) choroidal folds, (3) hyperopic refractive error shifts > 0.75 D, or (4) globe flattening^24,80^. Participants were labeled as showing signs of SANS (Table 1) if they developed at least one or more signs in either eye.

### Statistical methods

All statistical analyses were computed in R (R Foundation for Statistical Computing, Vienna, Austria, Version 1.3.1073) and JASP (University of Amsterdam, Amsterdam, Netherlands, Version 0.14.1) with the risk of type I error set at α = 0.05. The normality of data was assessed via Shapiro-Wilk tests. No correction was made for multiple comparisons in the described statistical analyses; this is a small sample study and should be considered exploratory. We report all individual *p* values and confidence intervals in tables^81,82^. Additionally, independent Student t-tests were applied to age and body mass index to test for differences between groups. All graphical representations were derived using GraphPad Prism 9 (GraphPad Software, La Jolla, CA, Version 9.4.0).

To assess the impact of HDBR + CO_2_ and hypercapnia on MV-PVS, five separate multivariate linear models were utilized to test for changes over time in the VAPeR group for each MV-PVS metric. We entered sex and mean-centered age as covariates into the full models to determine their influence. We were specifically interested in whether there were changes in MV-PVS metrics from pre-HDBR (timepoint 2) to in-HDBR (timepoints 3 and 4). For MV-PVS metrics showing significant changes from pre- to in-HDBR, we subsequently evaluated recovery by testing whether MV-PVS metric values at timepoints 5 and 6 remained different from the pre-intervention timepoint 2. We also conducted an exploratory analysis comparing the subset of participants (see Table 1) who developed at least one sign of SANS (SANS, *n* = 5) to the subset of participants who did not experience any signs of SANS (No-SANS, *n* = 6). Identical multivariate linear analyses were completed to test for differences in MV-PVS changes between SANS subgroups, as described previously. We also included sex and mean-centered age as covariates in these full models.

### Supplementary information

Supplementary Tables

## Data Availability

The data of this study are available via the NASA Life Sciences Data Archive.

## References

[1] Iliff JJ (2013). Brain-wide pathway for waste clearance captured by contrast-enhanced MRI. J. Clin. Invest..

[2] Iliff JJ (2012). A Paravascular Pathway Facilitates CSF Flow Through the Brain Parenchyma and the Clearance of Interstitial Solutes, Including Amyloid Beta. Sci. Transl. Med..

[3] Bacyinski A, Xu M, Wang W, Hu J (2017). The Paravascular Pathway for Brain Waste Clearance: Current Understanding, Significance and Controversy. Front Neuroanat..

[4] Troili F (2020). Perivascular Unit: This Must Be the Place. The Anatomical Crossroad Between the Immune, Vascular and Nervous System. Front Neuroanat..

[5] Nedergaard M, Goldman SA (2016). Brain Drain. Sci. Am..

[6] Ramirez J (2015). Visible Virchow-Robin spaces on magnetic resonance imaging of Alzheimer’s disease patients and normal elderly from the Sunnybrook Dementia Study. J. Alzheimers Dis..

[7] Potter GM, Chappell FM, Morris Z, Wardlaw JM (2015). Cerebral perivascular spaces visible on magnetic resonance imaging: development of a qualitative rating scale and its observer reliability. Cerebrovasc. Dis..

[8] Charidimou A (2014). White matter perivascular spaces: An MRI marker in pathology-proven cerebral amyloid angiopathy?. Neurology.

[9] Inglese M (2006). Clinical significance of dilated Virchow-Robin spaces in mild traumatic brain injury. Brain Inj..

[10] Patankar TF (2005). Dilatation of the Virchow-Robin Space Is a Sensitive Indicator of Cerebral Microvascular Disease: Study in Elderly Patients with Dementia. AJNR Am. J. Neuroradiol..

[11] Opel RA (2019). Effects of traumatic brain injury on sleep and enlarged perivascular spaces. J. Cereb. Blood Flow. Metab..

[12] Hupfeld KE (2022). Longitudinal MRI-visible perivascular space (PVS) changes with long-duration spaceflight. Sci. Rep..

[13] Barisano G (2022). The effect of prolonged spaceflight on cerebrospinal fluid and perivascular spaces of astronauts and cosmonauts. Proc. Natl. Acad. Sci. USA.

[14] Koppelmans V, Bloomberg JJ, Mulavara AP, Seidler RD (2016). Brain structural plasticity with spaceflight. NPJ Microgravity.

[15] Roberts DR (2017). Effects of Spaceflight on Astronaut Brain Structure as Indicated on MRI. N. Engl. J. Med..

[16] Lee JK (2019). Spaceflight-Associated Brain White Matter Microstructural Changes and Intracranial Fluid Redistribution. JAMA Neurol..

[17] Hargens AR, Vico L (2016). Long-duration bed rest as an analog to microgravity. J. Appl Physiol..

[18] Mulavara AP (2018). Physiological and Functional Alterations after Spaceflight and Bed Rest. Med Sci. Sports Exerc..

[19] Law J (2014). Relationship between carbon dioxide levels and reported headaches on the international space station. J. Occup. Environ. Med.

[20] Battisti-Charbonney A, Fisher J, Duffin J (2011). The cerebrovascular response to carbon dioxide in humans. J. Physiol..

[21] Zong X (2020). Morphology of perivascular spaces and enclosed blood vessels in young to middle-aged healthy adults at 7T: Dependences on age, brain region, and breathing gas. Neuroimage.

[22] Laurie SS (2019). Optic Disc Edema after 30 Days of Strict Head-down Tilt Bed Rest. Ophthalmology.

[23] Yang JW (2022). Spaceflight-associated neuro-ocular syndrome: a review of potential pathogenesis and intervention. Int J. Ophthalmol..

[24] Lee AG (2020). Spaceflight associated neuro-ocular syndrome (SANS) and the neuro-ophthalmologic effects of microgravity: a review and an update. NPJ Microgravity.

[25] Wostyn P, Killer HE, De Deyn PP (2017). Why a One-Way Ticket to Mars May Result in a One-Way Directional Glymphatic Flow to the Eye. J. Neuro-Ophthalmol..

[26] Mader TH (2021). Persistent Globe Flattening in Astronauts following Long-Duration Spaceflight. Neuroophthalmology.

[27] Wostyn P, Gibson CR, Mader TH (2022). The odyssey of the ocular and cerebrospinal fluids during a mission to Mars: the “ocular glymphatic system” under pressure. Eye (Lond.).

[28] Wostyn P, Mader TH, Gibson CR, Nedergaard M (2022). Does Long-Duration Exposure to Microgravity Lead to Dysregulation of the Brain and Ocular Glymphatic Systems?. Eye Brain.

[29] Zwart SR (2019). Association of Genetics and B Vitamin Status With the Magnitude of Optic Disc Edema During 30-Day Strict Head-Down Tilt Bed Rest. JAMA Ophthalmol..

[30] Zwart SR (2012). Vision changes after spaceflight are related to alterations in folate- and vitamin B-12-dependent one-carbon metabolism. J. Nutr..

[31] Smith SM, Zwart SR (2018). Spaceflight-related ocular changes: the potential role of genetics, and the potential of B vitamins as a countermeasure. Curr. Opin. Clin. Nutr. Metab. Care.

[32] Clement G (2022). Assessing the effects of artificial gravity in an analog of long-duration spaceflight: The protocol and implementation of the AGBRESA bed rest study. Front Physiol..

[33] Clément GR (2022). International standard measures during the AGBRESA bed rest study. Acta Astronautica.

[34] Tays GD (2022). The Effects of 30 min of Artificial Gravity on Cognitive and Sensorimotor Performance in a Spaceflight Analog Environment. Front. Neural. Circuits.

[35] Taoka T (2017). Evaluation of glymphatic system activity with the diffusion MR technique: diffusion tensor image analysis along the perivascular space (DTI-ALPS) in Alzheimer’s disease cases. Jpn J. Radio..

[36] Liu H (2021). Associations Among Diffusion Tensor Image Along the Perivascular Space (DTI-ALPS), Enlarged Perivascular Space (ePVS), and Cognitive Functions in Asymptomatic Patients With Carotid Plaque. Front Neurol..

[37] Taoka T (2022). Reproducibility of diffusion tensor image analysis along the perivascular space (DTI-ALPS) for evaluating interstitial fluid diffusivity and glymphatic function: CHanges in Alps index on Multiple conditiON acquIsition eXperiment (CHAMONIX) study. Jpn J. Radio..

[38] Lee JK (2021). Effects of Spaceflight Stressors on Brain Volume, Microstructure, and Intracranial Fluid Distribution. Cereb. Cortex Commun..

[39] Lee JK (2019). Head Down Tilt Bed Rest Plus Elevated CO2 as a Spaceflight Analog: Effects on Cognitive and Sensorimotor Performance. Front Hum. Neurosci..

[40] Berezuk C (2015). Virchow-Robin Spaces: Correlations with Polysomnography-Derived Sleep Parameters. Sleep.

[41] Barger LK (2014). Prevalence of sleep deficiency and use of hypnotic drugs in astronauts before, during, and after spaceflight: an observational study. Lancet Neurol..

[42] Jones CW, Basner M, Mollicone DJ, Mott CM, Dinges DF (2022). Sleep deficiency in spaceflight is associated with degraded neurobehavioral functions and elevated stress in astronauts on six-month missions aboard the International Space Station. Sleep.

[43] Buckey, J. C. in *Gravity and the Lung: Lessons from Microgravity* (eds G. K. Prisk, J. B. West, & M. Paiva) (Marcel Dekker, 2001).

[44] Buckey JC (2018). Microgravity-induced ocular changes are related to body weight. Am. J. Physiol.-Regulatory, Integr. Comp. Physiol..

[45] Laurie SS (2020). Unchanged cerebrovascular CO(2) reactivity and hypercapnic ventilatory response during strict head-down tilt bed rest in a mild hypercapnic environment. J. Physiol..

[46] Piantino J (2021). Link between Mild Traumatic Brain Injury, Poor Sleep, and Magnetic Resonance Imaging: Visible Perivascular Spaces in Veterans. J. Neurotrauma.

[47] Wu D (2020). Insulin Resistance Is Independently Associated With Enlarged Perivascular Space in the Basal Ganglia in Nondiabetic Healthy Elderly Population. Am. J. Alzheimers Dis. Other Demen.

[48] Martinez-Ramirez S (2013). Topography of dilated perivascular spaces in subjects from a memory clinic cohort. Neuology.

[49] Potter GM (2015). Enlarged perivascular spaces and cerebral small vessel disease. Int J. Stroke.

[50] Boespflug EL (2018). Targeted Assessment of Enlargement of the Perivascular Space in Alzheimer’s Disease and Vascular Dementia Subtypes Implicates Astroglial Involvement Specific to Alzheimer’s Disease. J. Alzheimers Dis..

[51] Gertje EC, van Westen D, Panizo C, Mattsson-Carlgren N, Hansson O (2021). Association of Enlarged Perivascular Spaces and Measures of Small Vessel and Alzheimer Disease. Neurology.

[52] Lynch M (2022). Perivascular spaces as a potential biomarker of Alzheimer’s disease. Front Neurosci..

[53] Taniguchi D, Shimura H, Watanabe M, Hattori N, Urabe T (2017). Widespread enlarged perivascular spaces associated with dementia and focal brain dysfunction: case report. BMC Neurol..

[54] Greenwald SH (2021). Intraocular pressure and choroidal thickness respond differently to lower body negative pressure during spaceflight. J. Appl Physiol. (1985).

[55] Taoka T, Naganawa S (2020). Glymphatic imaging using MRI. J. Magn. Reson Imaging.

[56] Meck JV, Dreyer SA, Warren LE (2009). Long-duration head-down bed rest: project overview, vital signs, and fluid balance. Aviat. Space Environ. Med..

[57] Cassady K (2016). Effects of a spaceflight analog environment on brain connectivity and behavior. Neuroimage.

[58] Marshall-Goebel K (2016). Effects of short-term exposure to head-down tilt on cerebral hemodynamics: a prospective evaluation of a spaceflight analog using phase-contrast MRI. J. Appl Physiol..

[59] Yuan P (2016). Increased Brain Activation for Dual Tasking with 70-Days Head-Down Bed Rest. Front Syst. Neurosci..

[60] Patel ZS (2020). Red risks for a journey to the red planet: The highest priority human health risks for a mission to Mars. NPJ Microgravity.

[61] Schwartz DL (2019). Autoidentification of perivascular spaces in white matter using clinical field strength T1 and FLAIR MR imaging. Neuroimage.

[62] McGregor HR (2023). Impacts of spaceflight experience on human brain structure. Sci. Rep..

[63] Buis, A. *The Atmosphere: Getting a Handle on Carbon Dioxide*, https://climate.nasa.gov/news/2915/the-atmosphere-getting-a-handle-on-carbon-dioxide/#:~:text=What%E2%80%99s%20in%20the%20Air%3F&text=By%20volume%2C%20the%20dry%20air,methane%2C%20nitrous%20oxide%20and%20ozone (2019).

[64] Hupfeld KE (2019). Neural Correlates of Vestibular Processing During a Spaceflight Analog With Elevated Carbon Dioxide (CO2): A Pilot Study. Front Syst. Neurosci..

[65] McGregor HR (2020). Brain connectivity and behavioral changes in a spaceflight analog environment with elevated CO2. Neuroimage.

[66] Salazar AP (2020). Neural Working Memory Changes During a Spaceflight Analog With Elevated Carbon Dioxide: A Pilot Study. Front Syst. Neurosci..

[67] Piantino J (2020). Characterization of MR Imaging-Visible Perivascular Spaces in the White Matter of Healthy Adolescents at 3T. AJNR Am. J. Neuroradiol..

[68] Smith SM (2002). Fast robust automated brain extraction. Hum. Brain Mapp..

[69] Leemans, A., Jeurissen, B. & Sijbers, J. in *17th Annual Meeting of Intl Soc Mag Reson Med*. (Hawaii, USA, 2009).

[70] Smith SM (2004). Advances in functional and structural MR image analysis and implementation as FSL. Neuroimage.

[71] Jenkinson M, Beckmann CF, Behrens TE, Woolrich MW, Smith SM (2012). Fsl. Neuroimage.

[72] Avants BB (2010). The optimal template effect in hippocampus studies of diseased populations. Neuroimage.

[73] Avants BB (2011). A reproducible evaluation of ANTs similarity metric performance in brain image registration. Neuroimage.

[74] Jenkinson M, Bannister P, Brady M, Smith S (2002). Improved Optimization for the Robust and Accurate Linear Registration and Motion Correction of Brain Images. NeuroImage.

[75] Tax, C. M. W., Vos, S. B. & Leemans, A. in *Diffusion Tensor Imaging* Ch. Chapter 7, 127–150 (2016).

[76] Vos SB (2017). The importance of correcting for signal drift in diffusion MRI. Magn. Reson Med..

[77] Perrone D (2015). The effect of Gibbs ringing artifacts on measures derived from diffusion MRI. Neuroimage.

[78] Leemans A, Jones DK (2009). The B-matrix must be rotated when correcting for subject motion in DTI data. Magn. Reson Med..

[79] Huang H (2008). Correction of B0 susceptibility induced distortion in diffusion-weighted images using large-deformation diffeomorphic metric mapping. Magn. Reson Imaging.

[80] Lee AG, Mader TH, Gibson CR, Tarver W (2017). Space Flight-Associated Neuro-ocular Syndrome. JAMA Ophthalmol..

[81] Saville DJ (1990). Multiple comparison procedures: The practical solution. Am. State.

[82] Rothman KJ (1990). No Adjustments Are Needed for Multiple Comparisons. Epidemiology.

