# Supplementary material for: Dynamic changes in perivascular space morphology predict signs of spaceflight-associated neuro-ocular syndrome in bed rest
Source: NPJ Microgravity. 2024 Mar 1;10:24. doi: 10.1038/s41526-024-00368-6 (PMC10907584; doi:10.1038/s41526-024-00368-6)
Supplement: Supplementary file 1 — Supplementary Tables [file 41526_2024_368_MOESM1_ESM.docx]

**Supplementary Tables**

**Dynamic Changes in Perivascular Space Morphology Predict Signs of Spaceflight-Associated Neuro-Ocular Syndrome in Bed Rest**

Sutton B. Richmond^a^, Rachael D. Seidler^a,b^, Jeffrey J. Iliff^c,d,e^, Daniel L. Schwartz^f,g^,

Madison Luther^h^, Lisa C. Silbert^f,i^, Scott J. Wood^j^, Jacob J. Bloomberg^j^, Edwin Mulder^k^,

Jessica K. Lee^a,k^, Alberto De Luca^l^, and Juan Piantino^h*^

^a^ Department of Applied Physiology and Kinesiology, University of Florida, 1864 Stadium Rd., Gainesville, FL, USA

^b^ Norman Fixel Institute for Neurological Diseases, University of Florida, Gainesville, FL, USA

^c^ Department of Psychiatry and Behavioral Sciences, University of Washington School of Medicine, Seattle, WA, USA

^d^ Department of Neurology, University of Washington School of Medicine, Seattle, WA, USA

^e^ VISN 20 Mental Illness Research, Education and Clinical Center (MIRECC), VA Puget Sound Health Care System, Seattle, WA, USA

^f^ Layton-NIA Oregon Aging and Alzheimer's Disease Research Center, Department of Neurology, Oregon Health & Science University, Portland, OR, USA

^g^ Advanced Imaging Research Center, Oregon Health & Science University, Portland, OR, USA

^h^ Department of Pediatrics, Division of Child Neurology, Doernbecher Children's Hospital, Oregon Health and Science University, Portland, OR, USA

^i^ Veteran's Affairs Portland Health Care System, Neurology, Portland, OR, USA

^j^ NASA Johnson Space Center, Houston, TX, USA

^k^ German Aerospace Center (DLR), Cologne, Germany

^l^ Department of Radiology, University Medical Center Utrecht, Utrecht, Netherlands

**Corresponding Author:** Juan Piantino, MD, MCR

Department of Pediatrics, Division of Child Neurology

Doernbecher Children's Hospital

707 SW Gaines St. CDRC-P

Portland, OR 97239

Email: piantino@ohsu.edu

**Supplementary Table 1.** **Mean ± Standard Deviation of the PVS and ALPS Index Metrics at Each Time Point**

|  | **HDBR+ CO_2_** | | |  | **HDBR (Controls)** |
| --- | --- | --- | --- | --- | --- |
| **Time Point** | **(*n = 11*)** | **SANS (*n = 5*)** | **No-SANS (n = 6)** |  | **(*n = 8*)** |
| **Total PVS volume (mm^3^/mm^3^ of WM) ^a^** | | | | | |
| Scan 1: Pre-HDBR, -13 days | 3.87e-4 ± 4.68e-4 | 4.63e-4 ± 5.27e-4 | 3.24e-4 ± 4.53e-4 |  | 4.20e-4 ± 4.69e-4 |
| Scan 2: Pre-HDBR, -7 days | 3.92e-4 ± 5.29e-4 | 3.51e-4 ± 3.93e-4 | 4.27e-4 ± 6.58e-4 |  | 5.22e-4 ± 5.62e-4 |
| Scan 3: HDBR, +7 days | 4.13e-4 ± 5.55e-4 | 4.12e-4 ± 4.63e-4 | 4.14e-4 ± 6.66e-4 |  | n/a |
| Scan 4: HDBR, +29 days | 4.42e-4 ± 5.10e-4 | 3.92e-4 ± 4.32e-4 | 4.81e-4 ± 6.13e-4 |  | 5.23e-4 ± 5.24e-4 |
| Scan 5: HDBR, +58 days | n/a | n/a | n/a |  | 3.67e-4 ± 3.42e-4 |
| Scan 6: Post-HDBR, R+7 days | 4.31e-4 ± 5.85e-4 | 4.45e-4 ± 5.73e-4 | 4.20e-4 ± 6.50e-4 |  | n/a |
| Scan 7: Post-HDBR, R+10 days | n/a | n/a | n/a |  | 4.51e-4 ± 4.46e-4 |
| Scan 8: Post-HDBR, R+13 days | 4.21e-4 ± 5.43e-4 | 4.43e-4 ± 5.92e-4 | 4.03e-4 ± 5.56e-4 |  | n/a |
| **Total PVS Number (No./mm^3^ of WM) ^a^** | | | | | |
| Scan 1: Pre-HDBR, -13 days | 3.68e-5 ± 4.13e-5 | 4.74e-5 ± 4.59e-5 | 2.79e-5 ± 3.90e-5 |  | 3.53e-5 ± 3.71e-5 |
| Scan 2: Pre-HDBR, -7 days | 3.67e-5 ± 4.41e-5 | 3.52e-5 ± 3.33e-5 | 3.80e-5 ± 5.47e-5 |  | 4.25e-5 ± 4.19e-5 |
| Scan 3: HDBR, +7 days | 3.88e-5 ± 4.79e-5 | 4.23e-5 ± 4.56e-5 | 3.60e-5 ± 5.38e-5 |  | n/a |
| Scan 4: HDBR, +29 days | 3.95e-5 ± 3.92e-5 | 3.83e-5 ± 3.30e-5 | 4.05e-5 ± 4.75e-5 |  | 4.48e-5 ± 3.70e-5 |
| Scan 5: HDBR, +58 days | n/a | n/a | n/a |  | 2.95e-5 ± 2.52e-5 |
| Scan 6: Post-HDBR, R+7 days | 4.13e-5 ± 5.30e-5 | 5.00e-5 ± 6.01e-5 | 3.41e-5 ± 5.09e-5 |  | n/a |
| Scan 7: Post-HDBR, R+10 days | n/a | n/a | n/a |  | 3.64e-5 ± 3.42e-5 |
| Scan 8: Post-HDBR, R+13 days | 4.21e-5 ± 5.06e-5 | 4.66e-5 ± 5.63e-5 | 3.84e-5 ± 5.04e-5 |  | n/a |
| **Median PVS Volume (mm^3^)** | | | | | |
| Scan 1: Pre-HDBR, -13 days | 9.39 ± 2.26 | 7.68 ± 0.75 | 10.81 ± 2.11 |  | 9.93 ± 2.28 |
| Scan 2: Pre-HDBR, -7 days | 9.26 ± 2.51 | 8.60 ± 1.04 | 9.81 ± 3.30 |  | 10.42 ± 3.01 |
| Scan 3: HDBR, +7 days | 8.38 ± 1.59 | 9.15 ± 1.22 | 7.73 ± 1.66 |  | n/a |
| Scan 4: HDBR, +29 days | 8.11 ± 1.10 | 7.97 ± 0.86 | 8.23 ± 1.35 |  | 9.00 ± 1.76 |
| Scan 5: HDBR, +58 days | n/a | n/a | n/a |  | 10.20 ± 1.35 |
| Scan 6: Post-HDBR, R+7 days | 9.32 ± 2.97 | 7.40 ± 0.84 | 10.92 ± 3.21 |  | n/a |
| Scan 7: Post-HDBR, R+10 days | n/a | n/a | n/a |  | 10.00 ± 1.30 |
| Scan 8: Post-HDBR, R+13 days | 8.58 ± 1.79 | 7.95 ± 0.76 | 9.10 ± 2.29 |  | n/a |
| **Median PVS Length (mm)** | | | | | |
| Scan 1: Pre-HDBR, -13 days | 6.72 ± 1.47 | 6.08 ± 0.93 | 7.26 ± 1.69 |  | 6.66 ± 0.60 |
| Scan 2: Pre-HDBR, -7 days | 6.35 ± 0.92 | 6.19 ± 0.63 | 6.48 ± 1.15 |  | 6.57 ± 1.06 |
| Scan 3: HDBR, +7 days | 6.54 ± 0.88 | 6.49 ± 0.89 | 6.58 ± 0.95 |  | n/a |
| Scan 4: HDBR, +29 days | 6.44 ± 0.76 | 6.41 ± 0.67 | 6.47 ± 0.90 |  | 6.43 ± 0.93 |
| Scan 5: HDBR, +58 days | n/a | n/a | n/a |  | 6.57 ± 0.51 |
| Scan 6: Post-HDBR, R+7 days | 6.72 ± 1.59 | 6.01 ± 0.65 | 7.31 ± 1.95 |  | n/a |
| Scan 7: Post-HDBR, R+10 days | n/a | n/a | n/a |  | 6.55 ± 0.94 |
| Scan 8: Post-HDBR, R+13 days | 6.60 ± 1.62 | 6.27 ± 0.94 | 6.88 ± 2.08 |  | n/a |
| **Median PVS Width (mm)** | | | | | |
| Scan 1: Pre-HDBR, -13 days | 2.74 ± 0.22 | 2.70 ± 0.13 | 2.78 ± 0.28 |  | 2.66 ± 0.27 |
| Scan 2: Pre-HDBR, -7 days | 2.72 ± 0.41 | 2.60 ± 0.06 | 2.82 ± 0.55 |  | 2.80 ± 0.19 |
| Scan 3: HDBR, +7 days | 2.55 ± 0.28 | 2.69 ± 0.17 | 2.44 ± 0.32 |  | n/a |
| Scan 4: HDBR, +29 days | 2.63 ± 0.26 | 2.55 ± 0.11 | 2.69 ± 0.34 |  | 2.77 ± 0.10 |
| Scan 5: HDBR, +58 days | n/a | n/a | n/a |  | 2.94 ± 0.28 |
| Scan 6: Post-HDBR, R+7 days | 2.63 ± 0.28 | 2.51 ± 0.31 | 2.72 ± 0.24 |  | n/a |
| Scan 7: Post-HDBR, R+10 days | n/a | n/a | n/a |  | 2.76 ± 0.22 |
| Scan 8: Post-HDBR, R+13 days | 2.70 ± 0.28 | 2.60 ± 0.20 | 2.78 ± 0.33 |  | n/a |
| **ALPS index (no unit)** | | | | | |
| Scan 1: Pre-HDBR, -13 days | 1.58 ± 0.21 | 1.56 ± 0.12 | 1.59 ± 0.12 |  | n/a |
| Scan 2: Pre-HDBR, -7 days | 1.57 ± 0.11 | 1.58 ± 0.14 | 1.57 ± 0.09 |  | n/a |
| Scan 3: HDBR, +7 days | 1.54 ± 0.16 | 1.60 ± 0.20 | 1.49 ± 0.11 |  | n/a |
| Scan 4: HDBR, +29 days | 1.49 ± 0.13 | 1.48 ± 0.16 | 1.50 ± 0.11 |  | n/a |
| Scan 5: Pre-HDBR, +37 days | 1.50 ± 0.08 | 1.53 ± 0.06 | 1.47 ± 0.09 |  | n/a |
| Scan 6: Pre-HDBR, +43 days | 1.45 ± 0.10 | 1.50 ± 0.12 | 1.40 ± 0.06 |  | n/a |

*Table S1 Note.* Here we report descriptive statistics for each PVS metric and ALPS index at all of the testing time points for the whole HDBR+ CO_2_ cohort, the SANS and no-SANS subgroups, and the HDBR control group. *HDBR* head-down tilt bed rest, *CO_2_* carbon dioxide, *SANS* spaceflight-associated neuro-ocular syndrome, *PVS* perivascular space, *WM* white matter, *ALPS* analysis along the perivascular space, *R* recovery, *n/a* not applicable.

**^a^** To account for individual differences in total brain tissue volumes, total PVS volume and number were normalized as follows: (total PVS volume (mm3) or number (No.)) / (total brain white matter volume (mm^3^), averaged across the two pre-bed rest time points).

**Supplementary Table 2.** **PVS Changes From Pre- to In-HDBR+CO_2_ and -** **HDBR**

| **Predictors** | **Value** | **Stand. Error** | **t-value** | ***p-value*** |
| --- | --- | --- | --- | --- |
| **Change in total PVS volume (mm^3^/mm^3^ of WM) ^a^** | | | | |
| (*intercept*) | 7.849 e^-4^ | 3.932 e^-4^ | 1.996 | 0.067 |
| *Time*: 29 Days into Bed Rest | 7.416 e^-7^ | 5.071 e^-5^ | 0.015 | 0.989 |
| *Group*: HDBR+CO_2_ (HDBR) | -7.656 e^-5^ | 2.823 e^-4^ | -0.271 | 0.791 |
| *Interaction*: Day 29 x SANS | 6.396 e^-6^ | 6.544 e^-5^ | 0.098 | 0.924 |
| **Change in total PVS number (No./mm^3^ of WM) ^a^** | | | | |
| (*intercept*) | 6.096 e^-5^ | 3.094 e^-5^ | 1.970 | 0.071 |
| *Time*: 29 Days into Bed Rest | 2.328 e^-6^ | 4.902 e^-6^ | 0.475 | 0.643 |
| *Group*: HDBR+CO_2_ (HDBR) | -1.925 e^-6^ | 2.225 e^-5^ | -0.086 | 0.932 |
| *Interaction*: Day 29 x SANS | -2.614 e^-6^ | 6.325 e^-6^ | -0.413 | 0.686 |
| **Change in median PVS volume (mm^3^)** | | | | |
| (*intercept*) | 11.146 | 1.331 | 8.371 | **< 0.001*** |
| *Time*: 29 Days into Bed Rest | -1.417 | 0.811 | -1.747 | 0.104 |
| *Group*: HDBR+CO_2_ (HDBR) | -1.137 | 1.026 | -1.109 | 0.288 |
| *Interaction*: Day 29 x SANS | 0.606 | 1.039 | 0.583 | 0.570 |
| **Change in median PVS length (mm)** | | | | |
| (*intercept*) | 7.079 | 0.579 | 12.223 | **< 0.001*** |
| *Time*: 29 Days into Bed Rest | -0.139 | 0.200 | -0.696 | 0.499 |
| *Group*: HDBR+CO_2_ (HDBR) | -0.155 | 0.425 | -0.364 | 0.721 |
| *Interaction*: Day 29 x SANS | 0.240 | 0.257 | 0.933 | 0.368 |
| **Change in median PVS width (mm)** | | | | |
| (*intercept*) | 2.754 | 0.247 | 11.162 | **< 0.001*** |
| *Time*: 29 Days into Bed Rest | -0.030 | 0.097 | -0.304 | 0.766 |
| *Group*: HDBR+CO_2_ (HDBR) | -0.103 | 0.182 | -0.565 | 0.582 |
| *Interaction*: Day 29 x SANS | 0.037 | 0.125 | 0.299 | 0.770 |

*Table S2 Note:* *p < 0.05; significant p values are **bolded**. Here we report the results of linear models testing whether the change in each PVS metric from pre- to in-bed rest differed significantly from 0. Our primary interest was whether the intercept was significant (p < 0.05), indicating a significant whole-group (HDBR+CO_2_ and HDBR**)** change in the PVS metric or ALPS index with bed rest. *HDBR* head-down tilt bed rest, *CO_2_* carbon dioxide, *SANS* spaceflight-associated neuro-ocular syndrome, *PVS* perivascular space, *WM* white matter, *ALPS* analysis along the perivascular space, *R* recovery.

**^a^** To account for individual differences in total brain tissue volumes, total PVS volume and number were normalized as follows: (total PVS volume (mm^3^) or number (No.)) / (total brain white matter volume (mm^3^), averaged across the two pre-bed rest time points).

**Supplementary Table 3.** **PVS changes from pre-bed rest to In-HDBR + CO_2_.**

| **Predictors** | **Value** | **Stand. Error** | **t-value** | ***p-value*** |
| --- | --- | --- | --- | --- |
| **Change in total PVS volume (mm^3^/mm^3^ of WM) ^a^** | | | | |
| (*intercept*) | 6.078 e^-4^ | 5.735 e^-4^ | 1.060 | 0.305 |
| *Time*: 7 Days into HDBR+CO_2_ | 1.340 e^-5^ | 5.892 e^-5^ | -0.228 | 0.823 |
| *Time*: 29 Days into HDBR+CO_2_ | 1.094 e^-5^ | 6.318 e^-5^ | -0.173 | 0.865 |
| *Group*: SANS (*No-SANS*) | 8.497 e^-5^ | 4.303 e^-4^ | -0.197 | 0.849 |
| *Interaction*: Day 7 x SANS | 7.477 e^-5^ | 8.739 e^-5^ | 0.856 | 0.405 |
| *Interaction*: Day 29 x SANS | 1.817 e^-5^ | 9.453 e^-5^ | 0.192 | 0.850 |
| **Change in total PVS number (No./mm^3^ of WM) ^a^** | | | | |
| (*intercept*) | 5.051 e^-5^ | 4.746 e^-5^ | 1.064 | 0.303 |
| *Time*: 7 Days into HDBR+CO_2_ | -1.986 e^-6^ | 6.498 e^-6^ | -0.306 | 0.764 |
| *Time*: 29 Days into HDBR+CO_2_ | -3.106 e^-6^ | 6.967 e^-6^ | -0.446 | 0.662 |
| *Group*: SANS (*No-SANS*) | -2.627 e^-6^ | 3.575 e^-5^ | -0.073 | 0.943 |
| *Interaction*: Day 7 x SANS | 9.062 e^-6^ | 9.638 e^-6^ | 0.940 | 0.361 |
| *Interaction*: Day 29 x SANS | 3.996 e^-6^ | 1.042 e^-5^ | 0.383 | 0.707 |
| **Change in median PVS volume (mm^3^)** | | | | |
| (*intercept*) | 10.843 | 1.091 | 9.940 | **< 0.001*** |
| *Time*: 7 Days into HDBR+CO_2_ | -2.083 | 0.963 | -2.163 | **0.046*** |
| *Time*: 29 Days into HDBR+CO_2_ | -1.269 | 1.024 | -1.239 | 0.233 |
| *Group*: SANS (*No-SANS*) | -2.038 | 1.084 | -1.879 | 0.102 |
| *Interaction*: Day 7 x SANS | 2.633 | 1.429 | 1.843 | 0.084 |
| *Interaction*: Day 29 x SANS | 0.361 | 1.524 | 0.237 | 0.816 |
| **Change in median PVS length (mm)** | | | | |
| (*intercept*) | 6.634 | 0.765 | 8.673 | **< 0.001*** |
| *Time*: 7 Days into HDBR+CO_2_ | 0.101 | 0.293 | 0.344 | 0.735 |
| *Time*: 29 Days into HDBR+CO_2_ | 0.169 | 0.314 | 0.538 | 0.598 |
| *Group*: SANS (*No-SANS*) | -0.728 | 0.611 | -1.191 | 0.115 |
| *Interaction*: Day 7 x SANS | 0.198 | 0.434 | 0.456 | 0.655 |
| *Interaction*: Day 29 x SANS | -0.186 | 0.469 | -0.397 | 0.696 |
| **Change in median PVS width (mm)** | | | | |
| (*intercept*) | 2.875 | 0.215 | 13.56 | **< 0.001*** |
| *Time*: 7 Days into HDBR+CO_2_ | -0.383 | 0.190 | -2.017 | 0.061 |
| *Time*: 29 Days into HDBR+CO_2_ | -0.099 | 0.202 | -0.492 | 0.629 |
| *Group*: SANS (*No-SANS*) | -0.294 | 0.214 | -1.374 | 0.212 |
| *Interaction*: Day 7 x SANS | 0.470 | 0.282 | 1.668 | 0.115 |
| *Interaction*: Day 29 x SANS | 0.026 | 0.301 | 0.085 | 0.933 |
| **ALPS index (no unit)** | | | | |
| (*intercept*) | 1.492 | 0.085 | 17.518 | **< 0.001*** |
| *Time*: 7 Days into HDBR+CO_2_ | -0.079 | 0.033 | -2.420 | **0.026*** |
| *Time*: 29 Days into HDBR+CO_2_ | -0.067 | 0.033 | -2.037 | 0.057 |
| *Group*: SANS (*No-SANS*) | 0.093 | 0.068 | 1.036 | 0.215 |
| *Interaction*: Day 7 x SANS | 0.100 | 0.049 | 2.060 | 0.054 |
| *Interaction*: Day 29 x SANS | -0.031 | 0.049 | -0.638 | 0.532 |

*Table S3 Note:* *p < 0.05; significant p values are **bolded**. Here we report the results of linear models testing whether the pre- to in-bed rest change in each PVS metric and ALPS index differed for the SANS vs. No-SANS participants. No-SANS served as the reference group (i.e., coded as = 0). Our primary interest here was whether there was a significant (p < 0.05) effect of SANS status on pre- to in-bed rest with elevated CO_2_ change in the PVS and ALPS index metrics. *HDBR* head-down tilt bed rest, *CO_2_* carbon dioxide, *SANS* spaceflight-associated neuro-ocular syndrome, *PVS* perivascular space, *WM* white matter, *ALPS* analysis along the perivascular space, *R* recovery.

**^a^** To account for individual differences in total brain tissue volumes, total PVS volume and number were normalized as follows: (total PVS volume (mm^3^) or number (No.)) / (total brain white matter volume (mm^3^), averaged across the two pre-bed rest time points).

**Supplementary Table 4.** **PVS changes from pre-HDBR + CO_2_ to recovery.**

| **Predictors** | **Value** | **Stand. Error** | **t-value** | ***p-value*** |
| --- | --- | --- | --- | --- |
| **Change in median PVS volume (mm^3^)** | | | | |
| (*intercept*) | 8.958 | 1.607 | 5.574 | **< 0.001*** |
| *Time*: R+7 Days | 2.859 | 0.921 | 3.105 | **0.007*** |
| *Time*: R+13 Days | 1.047 | 0.921 | 1.136 | 0.273 |
| *Group*: SANS (*No-SANS*) | 0.278 | 1.501 | 0.185 | 0.859 |
| *Interaction*: R+7 x SANS | -3.464 | 1.375 | -2.519 | **0.023*** |
| *Interaction*: R+13 x SANS | -1.101 | 1.375 | -0.801 | 0.435 |
| **ALPS index (no unit)** | | | | |
| (*intercept*) | 1.496 | 0.056 | 26.538 | **< 0.001*** |
| *Time*: R+7 Days | -0.097 | 0.051 | -1.906 | 0.073 |
| *Time*: R+13 Days | -0.165 | 0.051 | -3.227 | **0.005*** |
| *Group*: SANS (*No-SANS*) | 0.035 | 0.057 | 0.617 | 0.557 |
| *Interaction*: R+7 x SANS | 0.048 | 0.076 | 0.630 | 0.536 |
| *Interaction*: R+13 x SANS | 0.085 | 0.076 | 1.119 | 0.278 |

*Table S4 Note:* *p < 0.05; significant p values are **bolded**. Here we report the results of linear models testing whether the pre- to post-bed rest change in each PVS metric and ALPS index differed for the SANS vs. No-SANS participants. No-SANS served as the reference group (i.e., coded as = 0). Our primary interest here was whether there was a significant (p < 0.05) effect of SANS status on pre- to in-bed rest with elevated CO_2_ change in the PVS and ALPS index metrics. *HDBR* head-down tilt bed rest, *CO_2_* carbon dioxide, *SANS* spaceflight-associated neuro-ocular syndrome, *PVS* perivascular space, *WM* white matter, *ALPS* analysis along the perivascular space, *R* recovery.
